# Artificial intelligence for Amazonian health: validation is the price of entry

**DOI:** 10.1016/j.lana.2026.101627

**Published:** 2026-09-05

**Authors:** Matheus Serrao, Michella Lasmar, Luis Nakayama, Gustavo Monnerat, Leo Celi

**Affiliations:** a Laboratory for Computational Physiology, Massachusetts Institute of Technology, Cambridge, MA, USA; b Instituto de Ciências Matemáticas e de Computação, Universidade de São Paulo, São Carlos, SP, Brazil; c Universidade do Estado do Amazonas, Manaus, AM, Brazil; d Department of Ophthalmology, Federal University of São Paulo, São Paulo, SP, Brazil; e The Lancet Regional Health - Americas, Rio de Janeiro, RJ, Brazil; f Department of Biostatistics, Harvard T H Chan School of Public Health, Boston, MA, USA

At the *AI as Catalyst* meeting in Manaus (July 28–29, 2026; https://ia-como-catalisadora-manaus.netlify.app), participants ran a simple exercise. Working in teams, they wrote about ten clinical cases and put each one to widely used general-purpose chatbots twice: first in standard medical terminology, then in the terms and expressions a patient from the Amazon might use, with the same clinical facts, but carried by local language. The register of the answers changed with the framing, and so did their technical depth; more consequentially, the models inferred conditions from how the patient spoke rather than from what the case described. The lesson extends well beyond one workshop. Artificial intelligence (AI) tools built and validated elsewhere cannot be assumed safe or effective for Amazonian and Indigenous populations, and external validation grounded in local demography, language, and culture should become a precondition for clinical deployment in the region.

Of 691 AI-enabled medical devices cleared by the US Food and Drug Administration through mid-2023, over half did not report the size of the training sample and 95% reported no demographic representation; six reported randomised trial data and three reported patient outcomes.^1^ No comparable audit exists for devices authorised in Brazil, and the distance at which models fail is shorter than developers assume. An algorithm developed and tested entirely on Brazilian images reached 92·6% specificity in a statewide telehealth screening cohort but only 56·7% in a tertiary hospital cohort, with sensitivity near 95% in both.^2^ No Amazonian data have been tested, and the published gradient gives no reason to expect the decline to stop at the tertiary-hospital figure. The Amazon, with its distinct epidemiology, underrepresented genetic ancestries, and sparse digital records, is precisely where a model failing quietly could do so for years without detection.

However, demography is only half the problem. The Manaus exercise showed that language itself is a clinical variable. How a patient describes fever, pain, or fatigue is shaped by culture, and generative models interpret those descriptions through their training data, dominated by English and by biomedical registers; dialect alone has been shown to change the decisions language models make about the people who speak it.^3^ Brazil has 391 Indigenous peoples and 295 Indigenous languages,^4^ which are essentially absent from the corpora on which frontier models are trained. Validation must therefore test how patients speak and how clinicians document in the settings where a tool will actually be used, with local Portuguese and Indigenous-language descriptions of illness as core test material.

Validation alone will not settle who owns the data that makes it possible. In Indigenous communities, a single patient's record can describe dietary and social practices, territorial vulnerabilities, and traditional therapeutic knowledge that communities may not wish to circulate at all.^5^ Individual consent is insufficient to govern data that are collective by nature.

Brazilian law classifies health data as sensitive personal data, but its protections are attached to the individual,^6^ and collective instruments such as data trusts and data cooperatives remain under discussion, with none yet implemented at scale in Brazil. Meanwhile, the prevailing model of technology transfer repeats an old pattern: institutions arrive with ready-made tools, gather data to refine them, and leave little capacity behind. In Manaus this was named plainly: exporting raw health data and importing finished models is exporting raw material.

Therefore, the investment case is local, and the meeting offered proof of concept in both directions. Across an 18-hospital Brazilian network, adding training data from other regions usually reduced predictive performance, and training on a hospital's own patients was the best strategy at 11 of 18 sites.^7^ Manaus was an instructive exception: there the best results came from combining external data with local data, and re-tuning on local examples produced one of the largest gains recorded in the network (ADP Chiavegatto Filho, unpublished data, presented at the meeting). This underscores a broader structural principle: health innovations deliver optimal results when governance and execution are rooted locally. When antivenom treatment for snakebite was decentralised to Indigenous primary care with community assent, patients were 4·5 times more likely to receive antivenom within 6 h of the bite (95% CI 2·6–7·9) and presented with less severe envenoming on admission, with one death among 125 patients treated locally compared with five under the previous hospital-only model.^8^ Capacity is also human: South America's health workforce needs AI literacy now, not only to adopt these technologies, but to reduce the risk of cognitive reliance. Endoscopy data show that clinicians perform worse without AI after continuous exposure to it, with adenoma detection in standard colonoscopy falling from 28·4% to 22·4% after AI assistance was removed.^9^ Without deliberate training, trainees face the risk of never-skilling: they may reach practice without ever forming the judgement AI is meant to assist. Regional validation networks, led by Amazonian institutions with Indigenous communities as partners, would turn the region from a testing ground into a standard-setter.

AI will either widen or narrow health inequities in the Americas; nothing in the technology itself decides which. What decides is the evidence standard demanded before deployment,^10^ and who is in a position to demand it. That standard should be set in places like Manaus, not only to protect marginalised populations, but to redefine how health technologies are globally validated for complex, real-world environments.

## Contributors

All authors contributed to writing and checking the final draft, made the final decision to submit it, and are responsible for the content of the manuscript.

## Declaration of interests

GM works as an Editor for The Lancet Regional Health – Americas. All other authors declare no competing interests.
